# Value of the tuberculin skin testing and of an interferon-gamma release assay in haemodialysis patients after exposure to *M. tuberculosis*

**DOI:** 10.1186/1471-2334-12-195

**Published:** 2012-08-20

**Authors:** Luis Anibarro, Matilde Trigo, Diana Feijoó, Mónica Ríos, Luisa Palomares, Alberto Pena, Marta Núñez, Carlos Villaverde, África González-Fernández

**Affiliations:** 1 Unidade de tuberculose, Servizo de Medicina Interna, Complexo hospitalario de Pontevedra (CHOP). SERGAS, Mourente s/n, Pontevedra 36071, Spain; 2 Servizo de Microbioloxía, Complexo hospitalario de Pontevedra (CHOP). SERGAS, Mourente s/n, Pontevedra 36071, Spain; 3 Servizo de Nefroloxía, Complexo hospitalario de Pontevedra (CHOP). SERGAS, Mourente s/n, Pontevedra 36071, Spain; 4 Servizo de Neumoloxía, Complexo hospitalario de Pontevedra (CHOP). SERGAS, Mourente s/n, Pontevedra 36071, Spain; 5 Unidade de estatística. Universidade de Vigo, Campus de Lagoas, Marcosende, Vigo, Pontevedra 36310, Spain; 6 Área de Inmunoloxia, Centro de Investigaciones Biomédicas (CINBIO), Universidade de Vigo, Campus Lagoas Marcosende, Vigo, Pontevedra 36310, Spain

## Abstract

**Background:**

Patients with end-stage renal disease (ESRD) and *Mycobacterium tuberculosis* infection pose a high risk of developing active TB disease. It is therefore important to detect latent TB infection (LTBI) to be able to offer treatment and prevent progression to TB disease. We assessed the value of the tuberculin skin test (TST) and of an interferon-gamma release assay (Quantiferon®-TB Gold in-Tube, QFT) for diagnosing LTBI in ESRD patients, after prolonged exposure to a highly contagious TB case in a haemodialysis unit. As a high number of patients presented erythema without induration in the TST response, this type of reaction was also analysed.

**Method:**

The TST and QFT were simultaneously performed twelve weeks after the last possible exposure to a bacilliferous TB patient. If the first TST (TST-1) was negative, a second TST (TST-2) was performed 15 days later to detect a booster response. A comparison was made between the TST responses (including those cases with erythema without induration) and those for the QFT. The correlation with risk of infection and the concordance between tests were both analysed.

**Results:**

A total of 52 patients fulfilled the inclusion criteria. Overall, 11 patients (21.2%) had a positive TST response: 3 for TST-1 and 8 for TST-2, and 18 patients (34.6%) showed a positive QFT response (p = 0.065). Erythema without induration was found in 3 patients at TST-1 and in a further 9 patients at TST-2. The three patients with erythema without induration in TST-1 had a positive TST-2 response. Concordance between TST and QFT was weak for TST-1 (κ = 0.21); it was moderate for overall TST (κ = 0.49); and it was strong if both induration and erythema (κ = 0.67) were considered.

**Conclusions:**

In patients with ESRD, erythema without induration in the TST response could potentially be an indicator of *M. tuberculosis* infection. The QFT shows better accuracy for LTBI diagnosis than the TST.

## Background

Patients with end-stage renal disease (ESRD) have an increased risk for developing tuberculosis (TB) disease. It is estimated that once the infection is produced, the risk for developing active TB is 6 to 25 times higher than that in the general population [1,2]. It is difficult to diagnose TB disease in such cases, as there are frequently extrapulmonar locations and nonspecific symptoms. Moreover, they present a high mortality rate [3].

Patients with *Mycobacterium tuberculosis* infection after a recent exposure also have an increased risk for developing TB disease [4,5]. Hence, the risk of TB infection and disease is even higher in ESRD patients after a recent exposure to *M. tuberculosis*[6]. It is therefore crucial to detect latent TB infection (LTBI) in these patients and to offer early treatment that can prevent progression to active TB disease.

The Tuberculin Skin Test (TST) is based on a delayed-type hypersensitivity response against a purified protein derivative (PPD) from *M. Tuberculosis,* and has for many years been the standard tool for detecting LTBI. However, the value of this test is limited by its lack of specificity, due to cross-reactive immune responses caused by previous bacille Calmette-Guérin (BCG) vaccination, or by infection with non-tuberculous mycobacteria [7]. Moreover, TST has shown limited sensitivity for detecting *M. tuberculosis* infection in ESRD patients [8-12].

T-cell Interferon-gamma (IFN-γ) release assays, known as IGRAs, are emerging as new screening tools for the detection of *M. tuberculosis* infection. They incorporate specific antigens from *M. tuberculosis* to induce secretion of INF-γ as a marker of immune responses by T-cells. Such specific antigens are absent in the BCG strains and in the majority of non-tuberculous mycobacteria, avoiding antigenic cross-reactivity. IGRAs also incorporate an internal positive control, so that a failure of response may reflect an underlying anergy [13]. Two commercial tests are available: the Quantiferon®-TB Gold In-Tube (QFT) test (Cellestis Ltd, Carnegie, Australia), which uses ELISA to detect INF-γ in the culture supernatants, and the T-SPOT®.TB (Oxford Immunotec, Abindgdon, UK), which is based on the enzyme-linked inmunospot (ELISpot) assay.

As there is no gold-standard method for the diagnosis of LTBI, it is difficult to estimate the value of IGRA (and TST) for detecting asymptomatic *M. tuberculosis* infection. Correlation with the degree of exposure has been proposed as a surrogate marker of infection. In ESRD patients, IGRAs have shown a better correlation with risk factors for *M. tuberculosis* infection, and their use has recently been proposed instead of the TST in the British Thoracic Society Guidelines for prevention and management of TB infection and disease in patients with chronic kidney disease [14]. In addition, IGRAs show better correlation than TST in TB outbreaks in immunocompetent populations [15]. However, very few studies have directly compared the TST with an IGRA in haemodyalisis patients after a prolonged exposure to a bacilliferous patient [16,17].

After the notification of the diagnosis of a bacilliferous pulmonary TB case in a nurse working at a dialysis unit, we evaluated patients with ESRD who were attending the dialysis centre, with the TST and an IGRA test (QFT). The aims of the present study were to compare the results of an IGRA with those for the TST in patients with ESRD after a TB outbreak at the dialysis centre, as well as to identify factors associated with positive test results. The study also included an 18-month follow-up of the cohort of patients.

## Methods

### Description of the outbreak

A 52-year-old nurse working in a renal dialysis unit was diagnosed with bacilliferous pulmonary TB. Five months before diagnosis, the worker developed a cough and dysphonia. For nine weeks prior to diagnosis, the worker was off sick and she did not attend the work place. Therefore, she had no more contact with the patients attending the dialysis unit. Microscopic examination of sputum was 4+ positive of acid-fast bacilli, indicating infectiousness, and sputum culture yielded *M. tuberculosis* susceptible to all first-line anti-TB medications. The nurse had been working at the renal dialysis unit on week-long alternating morning and evening shifts. Each shift consisted of four hours of contact time with patients on haemodialysis treatment. The worker was considered to be infectious 17 weeks before she left her workplace [4]. All patients who attended the dialysis unit during this period were considered to be contacts and were evaluated. Twelve weeks after the last possible exposure to *M. tuberculosis*, initial screening was undertaken by simultaneously performing the tuberculin skin test (TST) and the QFT assay.

### Tuberculin skin test

Trained personnel performed the TST according to the Mantoux method following the standardized protocol: 0.1 mL (2 TU) of purified protein derivative of tuberculin RT 23 (Statens Serum Institute, Copenhagen, Denmark) was injected intradermally to the volar surface of the forearm. The TST results were read after 72 hours, and the maximum transverse diameter of the induration was estimated. TST values ≥ 5 mm were judged positive according to Spanish national guidelines [18]. A two-step TST was performed: if the first TST (TST-1) was negative (<5 mm induration), a second TST (TST-2) was conducted 15 days later to determine the development of a booster phenomenon. Patients without induration in the TST response, but with presence of erythema, were recorded as “erythema without induration”.

### Interferon-gamma release assay

Peripheral blood was processed for the IGRA, using the QuantiFERON®-TB Gold in-Tube Assay (QFT) according to the manufacturer’s instructions (Cellestis Ltd, Carnegie, Australia). One ml of whole blood was added to each of the three QFT tubes: TB antigen (containing ESAT-6, CFP-10 and TB7.7 antigens); mitogen positive control (containing phytohemagglutinin); and a negative control. Samples were frozen and stored at −70°C until analysis. The cut-off value for a positive test was 0.35 IU/mL as recommended by the manufacturer. Blood was collected for QFT immediately before TST-1, and before the start of the haemodialysis session.

### Data analysis

The following data were obtained: gender, age, body mass index (BMI), laboratory values (haemoglobin, urea, creatinine, albumin), BCG vaccination status, aetiology of renal disease, diabetes mellitus, immunosuppressive therapy, weeks of exposure to the index case, and the presence of old healed TB in chest radiography. BCG vaccination status was evaluated by the careful revision of the BCG scar by an expertise nurse or by the BCG vaccination certificate.

### Follow-up

Patients with either a positive TST or a positive QFT response were considered for treatment for LTBI after active TB disease was excluded. No cases of active TB were found during the study period. The possibility of TB symptoms was carefully monitored throughout the long-term 18-month follow-up period.

### Statistical analyses

Unless otherwise indicated, data are presented as mean ± standard deviation (SD). Associations between test results and variables were assessed by Pearson’s *χ*^2^-square test or Fisher’s exact for categorical variables, and with Student’s *t* test or Mann-Witney U rank test for continuous variables. All statistically significant factors ( *P*<0.05) or clinically relevant factors with a *P*<0.2 determined by univariate analysis were included in models of multivariate logistic regression analysis. Comparison between test results was performed using the McNemar test. All reported *P* values were two-sided. Concordance between TST and QFT was assessed using the Kappa coefficient (κ). Kappa values indicate weak (≤ 0.40), moderate (0.41-0.60), strong (0.61-0.80), or excellent (>0.80) agreement. The SPSS statistical software for Windows (SPSS version 15.0; SPSS Inc, Chicago, IL, USA) was used in the analyses.

Institutional ethical approval was obtained from the Ethical Committee of Clinical Research (*Xunta de Galicia*, Spain) and written informed consent was obtained from the index case.

## Results

### Characteristics of the contacts

Initially, the contact investigation included 58 patients with ESRD attending the haemodialysis unit during the 17-week infectious period of the TB index case. One patient refused to be included in the study, and a previous positive TST was documented for five other patients, who were also excluded from further analysis. Hence, a total of 52 patients were included in the study.

The mean age of these patients was 62 ±16.8 years. Seven patients (13.5%) had been vaccinated with BCG. The aetiology of the end-stage renal failure was hypertensive nephrosclerosis (31%), diabetic nephropathy (19%), others (29%), and unknown (21%). Eight patients were receiving immunosuppressive therapy. Two patients recalled previous contact with a TB patient, but both had had a negative TST result.

Each patient attended the dialysis ward for about 4 hours for three days a week, except for one patient who had dialysis on a daily basis. A total of 46 patients (88.5%) had been in contact with the index case for the whole 17-week infectious period. The control unit (where the health personnel work) is located at the centre of the room, so the degree of exposure to the index case was considered to be almost identical for all patients. The other 6 patients with a shorter period of exposure had been in contact with the index case for 4, 6, 11, 12 (two patients) and 13 weeks each.

Other demographic, clinical characteristics and laboratory findings are shown in Table 1.

**Table 1:** Basic demographic characteristics of 52 patients with end-stage renal disease from a haemodialysis unit, all contacts of a healthcare worker who had active pulmonary tuberculosis while working at the dialysis centre

**CHARACTERISTICS**	**STUDY GROUP**
Age in years, mean ± SD (range)	62 ± 16.8 (24 to 89)
Male gender, n (%)	31 (59.6)
BCG vaccination, yes, n (%)	7 (13.5)
BMI, mean ± SD	27.1 ± 5.0
Hemoglobin (mg/dL), mean ± SD	11.5 ± 1.4
Albumin level (mg/dL) mean ± SD	2.8 ± 0.4
Diabetes mellitus, yes, n (%)	8 (15.4)
Exposure to index case (weeks, %)	
17 week	46 (88.5)
< 17 weeks	6 (11.5)
Radiographic evidence of healed TB, n (%)	5 (9.6)

*BMI:* Body mass index.

Infectiousness of the Index Case was estimated for 17 weeks before last exposure.

### TST and QFT results

#### TST results

**Figure 1:**
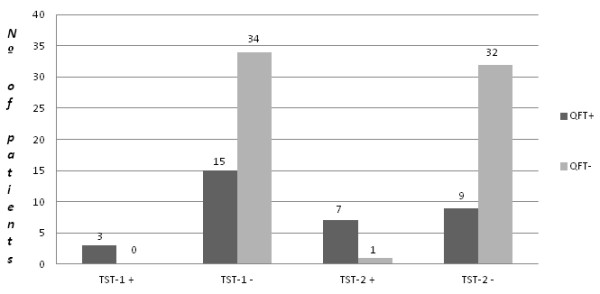
**Results of the Tuberculin Skin Test and of the Quantiferon®-TB Gold in-Tube assay.** TST-1: Tuberculin skin test result in the first-step testing. TST-2: Tuberculin skin test result in the second-step testing (15 days after TST-1). QFT: Quantiferon®-TB Gold in-tube assay.

Of the 52 patients who had been in contact with the index case, 3 (5.8%) had a positive TST result for the first test (TST-1) (Figure 1). Fifteen days later, the remaining 49 patients had a second TST (TST-2) and the TST-2 was positive in 8 patients (16.3%). Overall, 11 patients (21.2%) had a final positive TST. In BCG-vaccinated individuals, the TST was positive in only 1 patient, and this was detected at TST-2. In the five patients with radiographic findings suggesting possible old healed TB, the TST was positive in three cases (one at TST-1 and the other two at TST-2). The induration measurement of tuberculin reaction was >10 mm in every patient with a positive result.

Three patients (6.1%) out of 49 with a negative TST-1 only had erythema. The frequency of patients showing erythema without induration even increased at the second TST, with it being observed in 9 out of the 41 patients with a negative TST-2 (22.0%). It is worth noting that for the three patients with erythema but a negative TST-1, the second TST performed 15 days later was clearly positive (Table 2).

**Table 2:** Relationship between the tuberculin skin test results and erythema

	**TST (+)**	**TST(−) erythema (+)**	**TST (−) erythema (−)**
**TST-1**	3	3*	46
**TST-2**	8	9	32

*TST-1*: Tuberculin skin test result in the first-step testing.

*TST-2*: Tuberculin skin test result in the second-step testing (15 days after TST-1).

* The three patients with a negative TST-1 result and erythema boosted to a positive TST-2 result.

A positive TST result was not associated with any of the variables analysed, although the presence of an old healed TB in chest radiography approached statistical significance (p = 0.057).

#### QFT results

The QFT was performed at the same time as the TST-1 in all the 52 patients, and it was positive in 18 cases (34.6%). No indeterminate results were found. In BCG-vaccinated individuals, the QFT result was positive in only one patient, who had both negative TST-1 and TST-2 results. In the five patients with radiographic findings suggesting possible old healed TB, QFT was positive in three cases (all with positive TST results) and it was negative in the other two patients, who also had a negative TST result. A positive QFT result was only significantly associated with the presence of old healed TB in chest radiography (p = 0.043).

Agreement between the TST and QFT results was found to be weak when taking into account only the initial TST (κ = 0.21), whereas it was moderate when both TST results (TST-1 and TST-2) were considered (κ = 0.49) (Table 3).

**Table 3:** Agreement between the results of tuberculin skin testing with those for the Quantiferon®-TB Gold in-tube assay

			**TST-1**			**Overall TST**		
			**negative**	**positive**	**Total**	**negative**	**positive**	**Total**
**QFT**	negative	n	34	0	**34**	32	2	**34**
		(%)	65.4%	0%	**65.4%**	61.5%	3.8%	**65.4%**
	positive	n	15	3	**18**	9	9	**18**
		(%)	28.8%	5.8%	**34.6%**	17.3%	17.3%	**34.6%**
	Total	n	49	3	**52**	41	11	**52**
		(%)	94.2%	5.8%	**100%**	78.8%	21.2%	**100%**
*kappa value*				*0.21*			*0.49*	

*TST-1:* Tuberculin skin test result in the first-step testing.

*Overall TST:* Tuberculin skin test result after TST-1 and second-step testing (15 days after first testing).

*QFT:* Quantiferon®-TB Gold in-tube assay.

Overall, the QFT was positive in 18 patients (34.6%), while the TST was positive in 11 patients (21.2%) (p = 0.065).

### Value of erythema without induration in the TST response

As an unexpectedly high number of patients presented erythema without any induration in the TST response, we attempted to verify if erythema alone could be a valuable marker for LTBI. Of the 12 patients with erythema and no induration in the TST, 8 had a positive QFT result (2 out of 3 cases with a negative TST-1 result and 6 out of 9 cases with a negative TST-2 result). If both responses (the presence of erythema without induration or a positive TST result) were considered to be positive test results, agreement between the QFT and TST improved to κ = 0.67 (strong agreement), as shown in Table 4.

**Table 4:** Agreement between the results of tuberculin skin testing and presence of erythema with those for the Quantiferon-TB Gold in-tube assay

			**TST/Erythema**		
			**TST (−) Erythema (−)**	**TST (+) or erythema (+)**	**Total**
**QFT**	**negative**	**n**	29	5	**34**
		**%**	55.8%	9.6%	**65.4%**
	**positive**	**n**	3	15	**18**
		**%**	5.8%	28.8%	**34.6%**
	**total**	**n**	32	20	**52**
		**%**	61.5%	38.5%	**100`**
***kappa value***			***0.67***		

*TST:* Tuberculin Skin Test.

*QFT:* Quantiferon®-TB Gold in-Tube assay.

### Contact follow-up

All contacts with a positive result for either the TST or the QFT were considered to have LTBI, after active TB was excluded. Preventive treatment with isoniazid for 9 months was considered for these patients.

Twenty patients (38.5%) were considered to have LTBI (two on the basis of a positive TST result, nine on the basis of a positive QFT result, and nine with positive results for both TST and QFT). Preventive treatment was started for 11 patients, of which 8 patients completed the treatment. Isoniazid treatment was stopped in two patients because of toxicity, and the final patient in this group died during LTBI treatment, from causes unrelated to either TB or isoniazid toxicity. The remaining 9 patients did not start LTBI treatment: four of them due to poor basal performance, while five declined to receive the treatment.

Eighteen months after the outbreak, none of the 20 patients with LTBI developed active TB. Five patients died during the follow-up period (one during LTBI treatment and four patients without treatment). No death was related to TB.

The remaining 32 patients with negative results for both QFT and TST were considered to be not infected, and did not receive LTBI treatment. After the 18-month follow-up period of close clinical monitoring, no case of active TB was found among this group. Four patients died during the follow-up period, none of the deaths were related to TB.

## Discussion

The present study assessed the value of TST and QFT in haemodialysis patients after prolonged exposure to a highly contagious pulmonary TB patient. The results obtained showed three major findings. First, a single TST has low sensitivity in detecting LTBI and a two-step strategy must be carried out to gain sensitivity. Second, erythema alone (without induration) may be a valuable marker when interpreting TST in haemodialysis patients. And third, the QFT shows better sensitivity than the TST in detecting LTBI in haemodialysis patients.

TST has been shown to be unreliable in patients with advanced chronic kidney disease, and although a positive test may be useful for LTBI diagnosis, a negative one cannot be assumed to be a true negative [8,10,11]. Uraemia is a well-known risk factor for impaired immune cellular response [9,19], and this fact is decisive for both a lower sensitivity of the TST and a higher risk of progression from LTBI to TB disease [12]. It has been shown that immune cells can be activated in ESRD patients, but the functionality of these cells is impaired. This is especially true for the subpopulation of T helper 1 (TH1) lymphocytes, which are the cells mainly responsible for cellular immunity and delayed hypersensitivity responses. Moreover, there is a decrease in the number of B cells in a pro-inflammatory environment (complement activation, inflammatory cytokines), together with an imbalance in the ratio of TH1/TH2 lymphocytes [20]. The suppression of the immune TH1 status entails a higher rate of anergy, eventually leading to lower positive TST responses compared to the QFT results. Although limited by the absence of a gold-standard method to confirm LTBI, in our study QFT has shown a higher response than TST, which approaches statistical significance.

Two-step testing in TST has been shown to be crucial for LTBI diagnosis in patients with ESRD, in the absence of a known recent exposure to a contagious index case [21,22]. In our study, 8 patients with an initially negative TST had a second positive TST 15 days later; thereby, highlighting the importance of the booster effect in ESRD patients. The booster effect is believed to result from recall of waned cell-mediated immunity, akin to the anamnestic response. A first tuberculin test may boost cell-mediated immunity in patients with otherwise impaired immune response, such as patients with ESRD [23]. Several studies have addressed the booster phenomenon in ESRD patients without known recent exposure to a TB index case [21,22,24-26]. Cengiz et al. detected a boosted response in a second TST, performed seven days after a first TST, in 24.3% of patients on haemodialysis [22]. Similarly, Habesglu et al. found the booster phenomenon in 29.7% of patients undergoing haemodialysis treatment and with no epidemiologically recognised factors for LTBI [25]. Our study has addressed the booster response in the context of a recent exposure to a highly contagious TB index case. Therefore, it could be difficult to distinguish between a booster effect from a real conversion after the tuberculin “window period”. Nevertheless, this is very unlikely, because both TST and QFT were performed 12 weeks after the last possible exposure to the Index Case, which would make very unlikely a conversion attributable to the “window period” [27]. Our findings support the view that two-step TST must be performed in patients with ESRD, even in the context of a recent exposure to *M. tuberculosis.*

An unexpected finding of our study was the high number of patients with erythema, but without induration, in the TST responses. Erythema without induration is not generally considered in the interpretation of the TST in most International Guidelines. However, some Japanese guidelines suggest taking erythema without induration into consideration when the measurement is over 20 mm [28]. In a contact tracing study carried out with 566 BCG-vaccinated school students in Japan, the erythema measurement was correlated with both the induration measurement and the degree of exposure to the index case*.* These results suggest that erythema (irrespective of the induration response) could have a significant value in the interpretation of the TST results. However, in this study, no concomitant IGRA testing was performed [29]. In our study, joint assessment of induration and erythema has shown better correlation with the QFT results than when only induration was taken into account in the TST response. All 3 patients with erythema, but with a negative result in the initial TST-1, developed a positive TST response 15 days later. Moreover, of the 9 patients with erythema without induration in the second TST, six (66.7%) had a positive QFT result. Overall, our results suggest that in patients with impaired cell-mediated immune response, such as patients with ESRD, erythema without induration could be indicative of a weak immune response to *M. tuberculosis* antigens, even though it did not yield skin induration. Our findings indicate that *M. tuberculosis* infection could be considered in patients with ESRD and with no induration erythema shown in the TST response, particularly in situations of high epidemiological risk of infection. Nevertheless, more studies and a higher number of patients than those presented in our study are required to fully verify this statement.

As there is no gold-standard method to confirm diagnosis of LTBI, the accuracy of tests for evaluating LTBI relies on indirect evidence of infection. There is increasing evidence that IGRA results have a better correlation with known risk factors for LTBI than the TST responses [13]. In addition, several studies have assessed this issue in patients with chronic kidney disease, indicating that for these groups of patients, IGRAs have a greater accuracy than TST in the diagnosis of LTBI [30-34]. However, only a few studies have compared TST and IGRA results in haemodialysis patients after a recent exposure to a bacilliferous TB patient [16,17]. Winthrop et al. found a better correlation with risk exposure using Quantiferon-TB-Gold® test (an older generation of QFT) than when using the TST after exposure to a pulmonary TB patient in a haemodialysis centre [16]. Yet, they found that the rates of positive results using the TST and the IGRA were not significantly different, whereas we found a value close to statistical significance (p = 0.065). The conclusion derived from the above cited study and from our current findings (using the new generation of QFT) is that IGRAs may offer greater accuracy for the diagnosis of LTBI in ESRD patients, after exposure to an infectious TB patient [14].

Our study has several limitations that require further comments. First, due to the limited number of patients included in the study, our results must be interpreted with caution, and larger series of patients are necessary to confirm our findings. This is especially true for the value of erythema, as very few studies have previously assessed this item. Second, as there is no gold-standard method for diagnosing true LTBI, it cannot be ruled out that higher positivity rates for the QFT could include some false positive results. Nevertheless, there is increasing evidence about the value of an IGRA as a marker for LTBI, and even as a marker for the risk of progression to active TB [35]. Finally, only one commercial IGRA (QFT) has been evaluated in our study. It has been suggested that the other commercially available IGRA (T-SPOT.TB®) may have an improved sensitivity in patients with immunodeficiency disorders [36]. There are several previous studies that have compared different commercially available IGRAs in ESRD patients [30,32,33]. In a Korean study comparing the performance of TST, QFT and T-SPOT.TB in patients undergoing haemodialysis, the positive rates were 23.5%, 45.9% and 60.4% respectively, suggesting a higher sensitivity for the T-SPOT.TB test. In addition, the frequency of indeterminate results was higher for the QFT compared with the T-SPOT.TB test [32]. In contrast, two other studies failed to demonstrate significantly better sensitivities with the T-SPOT.TB test than with the QFT: 47% vs. 40%, respectively, using an older generation of QFT in a Taiwanese study [30], or 22% vs. 46%, respectively, in 62 Swiss patients deemed to have LTBI [33].

## Conclusions

Our results indicate that the QFT shows better sensitivity than the TST in detecting LTBI in haemodialysis patients, after exposure to *M. tuberculosis*. Nevertheless, if the TST is used for detecting LTBI, a single TST result has low sensitivity and a two-step strategy must be performed to gain sensitivity.

Although the relatively small number of patients included in the present study limits, to some extent, the significance of the conclusions, it can still be emphasized that special care should be taken in patients with ESRD, who show erythema after the TST, because erythema alone (without induration) may be a marker of LTBI when interpreting TST in these patients.

## Consent statement

Institutional ethical approval for the study was obtained from the Ethical Committee of Clinical Research (*Xunta de Galicia*, Spain) and written informed consent for publication was obtained from the index case. A copy of the written consent is available for review by the Series Editor of the journal.

## Competing interests

The authors declare that they have no competing interests.

## Authors’ contributions

LA provided patient care and was responsible for data management, design of the study and drafting of the manuscript. MT and AP performed laboratory tests and were involved in writing the draft version. DF, MR, LP and MN made substantial contributions to the acquisition of data, participated in recruitment of subjects, provided patient care and were involved in writing the draft version. CV performed the statistical analysis. AGF revised the draft carefully for important intellectual content. All authors read and approved the final version of the manuscript.

## Pre-publication history

The pre-publication history for this paper can be accessed here:

http://www.biomedcentral.com/1471-2334/12/195/prepub
